# From neglect to necessity: the role of innate immunity in cutaneous squamous cell carcinoma therapy

**DOI:** 10.3389/fimmu.2025.1570032

**Published:** 2025-04-25

**Authors:** Yong He, Ting Tian, Yuancheng Li, Yong Zeng, Xiaoke Wang, Leqi Qian, Tian Tian, Mingjun Jiang, Liming Li

**Affiliations:** ^1^ Hospital for Skin Diseases, Institute of Dermatology, Chinese Academy of Medical Sciences & Peking Union Medical College, Nanjing, China; ^2^ Hunan Key Laboratory of Medical Epigenomics, Department of Dermatology, The Second Xiangya Hospital of Central South University, Changsha, China; ^3^ Key Laboratory of Basic and Translational Research on Immune-Mediated Skin Diseases, Chinese Academy of Medical Sciences, Nanjing, China

**Keywords:** innate immune cells, cutaneous squamous cell carcinoma, immunotherapy, macrophages, neutrophils

## Abstract

As the second most common non-melanoma skin cancer, cutaneous squamous cell carcinoma (cSCC) has experienced a significant increase in incidence. Although clinical detection is relatively easy, a considerable number of patients are diagnosed at an advanced stage, featuring local tissue infiltration and distant metastasis. Cemiplimab, along with other immune checkpoint inhibitors, enhances T cell activation by blocking the PD-1 pathway, resulting in notable improvements in clinical outcomes. Nonetheless, approximately 50% of the patients with advanced cSCC remain unresponsive to this therapeutic approach. It emphasizes the importance of finding innovative therapeutic targets and strategies to boost the success of immunotherapy across a wider range of patients. Therefore, we focused on frequently neglected functions of innate immune cells. Emerging evidence indicates that innate immune cells exhibit considerable heterogeneity and plasticity, fundamentally contributing to tumor initiation and development. The identification and eradication of cancer cells, along with the modulation of adaptive immune responses, are essential roles of these cells. Consequently, targeting innate immune cells to activate anti-tumor immune responses presents significant potential for enhancing immunotherapeutic strategies in cSCC.

## 1 Introduction

Cutaneous squamous cell carcinoma (cSCC) is a type of skin cancer that develops from the abnormal growth of keratinocyte (KC). It is the second most common non-melanoma skin cancer worldwide, with around 2 million new cases annually (1, 2). cSCC is one of the fastest-growing skin cancer types, but its actual incidence rates may be underreported. This underreporting is frequently seen in national cancer registries because they fail to record cSCC or only note the initial tumor (3). Ultraviolet (UV) radiation, particularly UVB and UVA, is linked to a heightened risk of developing skin cancer. This connection highlights the necessity of protecting the skin from overexposure to sunlight, as the harmful effects of these types of radiation can lead to severe health consequences. Other established risk factors encompass immunosuppression, human papillomavirus infection, a history of cSCC, ionizing radiation exposure, advanced age, chronic ulcers, burn wounds, persistent scars, and pre-existing chronic skin conditions like dystrophic epidermolysis bullosa and erosive lichen planus (4). After the initial tumor has been surgically removed, the likelihood of local recurrence or metastasis for cSCC is about 4%. The metastatic rate among immunosuppressed individuals, especially solid organ transplant recipients, is twice as high (1, 5). In advanced stages, the prognosis is unfavorable, and half of the patients survived fewer than 2 years (6). Potentially curative treatment options for localized cSCC include surgery, photodynamic therapy, radiation therapy, and topical immunotherapy, which can cause significant morbidities such as pain, ulceration, and disfigurement, and affect the quality of life (1, 7). Systemic therapies, including platinum-based chemotherapies and inhibitors of the epidermal growth factor receptor (like cetuximab), may be utilized for advanced cSCC. Recently, there has been a significant advancement in the field with the approval of the first immune checkpoint inhibitor (ICI) that specifically targets PD-1, known as cemiplimab. This innovative treatment has shown promising results, achieving an overall response rate of approximately 50% when administered as a first-line therapy for patients (8). Besides, a recent study highlights the synergistic potential of combining PD-1 inhibitors (e.g., cemiplimab) with radiotherapy, enhancing both local and distant tumor control through T-cell-dependent mechanisms (9). Cemiplimab exerts its effects by inhibiting immunosuppressive signals in T-cells, thereby activating the tumor-killing potential of the adaptive immune system (10). However, many patients are reluctant to receive these immunotherapies, and the long-term survival impact of these ICIs on metastatic cSCC remains under investigation, with current five-year survival estimates below 30% (4).

The innate immune system is a crucial component of defending the host, functioning as the first line of protection against infections. It comprises both physical and chemical barriers that help prevent microbial invasion, alongside various specialized cell types that are responsible for recognizing a wide range of microorganisms based on conserved patterns (11). This system is crucial for sustaining the body’s defenses, identifying, and responding to various pathogens. Key components in the innate immunity include macrophages, neutrophils, dendritic cells (DCs), myeloid-derived suppressor cells (MDSCs), and so on (12). These cells not only initiate a response to infections but also essential in activating adaptive immunity. For instance, macrophages engage in phagocytosis to engulf pathogens, while NK cells exhibit natural cytotoxicity to eliminate infected or malignantly transformed cells. Moreover, the innate immune system contributes to adaptive immunity through processes like antibody-dependent cell cytotoxicity and enhanced phagocytosis. This is facilitated by the presence of Fc receptors (FcRs) on macrophages and NK cells, which mediate interactions with antibodies bound to pathogens or infected cells (13). Notably, the activation of innate immunity may, counterintuitively, aid in tumor progression. The innate immune cells present within tumors exhibit heterogeneity and plasticity (14), with their phenotypes and functions evolving in response to changes in the local environment. Depending on interactions with other cells or tumors and the soluble factors available in the microenvironment, innate cells can develop both pro- and anti-tumor properties (15). Growing evidence indicates that immune cells displaying immunosuppressive characteristics can still preserve their anti-tumor capabilities, and successful strategies may promote their reprogramming to an anti-tumor profile (10). Consequently, innate immune cells and their relevant regulatory targets present promising targets for enhancing the anti-tumor responses or serve as complementary strategies to ICIs in therapy (16). Therefore, developing a comprehensive understanding of innate immune cells is essential to improve current immunotherapy performance and increase the likelihood of favorable patient outcomes.

Lately, single-cell RNA sequencing (scRNA-seq) and spatial transcriptome sequencing have reignited interest in how innate immunity contributes to cancer progression. Recent advancements enable researchers to categorize cancers by their immune composition, considering the types, phenotypes, and spatial distribution in the tumor microenvironment (TME). Furthermore, researchers have emphasized the adaptability of these cells, specifically how their characteristics and roles shift according to the surrounding environment. In this article, we summarize the current insights into the roles that innate immune cells may play in cSCC, as well as relevant preclinical studies utilizing innate immunity as a therapeutic strategy.

## 2 The role of innate immune cells in cSCC development

### 2.1 Macrophages

Macrophages exist in every tissue and are vital for maintaining balance and managing diseases (17). They act as the initial defense against infections and tissue damage by engulfing pathogens and cell debris (18, 19). Tumor-associated macrophages (TAMs) are macrophages found in the TME that are typically implicated in angiogenesis, metastasis, tumor initiation, and tumor development (20). However, TAMs can also exhibit tumouricidal functions by enhancing phagocytic activity and pro-inflammatory responses to suppress tumor growth (21). Notably, TAMs can play dual roles depending on the context. Classically activated macrophages (M1) and alternatively activated macrophages (M2) are two different subtypes of TAMs that exhibit extraordinary plasticity. M1 macrophages are distinguished by high major histocompatibility complex class II (MHCII) expression and low mannose receptor (CD206) expression (22). By releasing cytokines that promote inflammation such as Interleukin (IL)-12, IL-23, and tumor necrosis factor (TNF)-α, as well as nitric oxide (NO), these macrophages contribute to activate immune system by preventing the growth and proliferation of cancer cells (19). Conversely, M2 macrophages (CD163^+^) are distinguished by their elevated mannose receptor and reduced MHC class II levels. They can be activated by anti-inflammatory cytokines like IL-4, IL-10, or IL-13 (23). M2 macrophages release immunosuppressive cytokines, including vascular endothelial growth factor (VEGF), arginase-1 (Arg-1), transforming growth factor-β (TGF-β) and IL-10, thereby contributing to tumor progression by facilitating angiogenesis, invasion, and migration (24).

**Figure 1:**
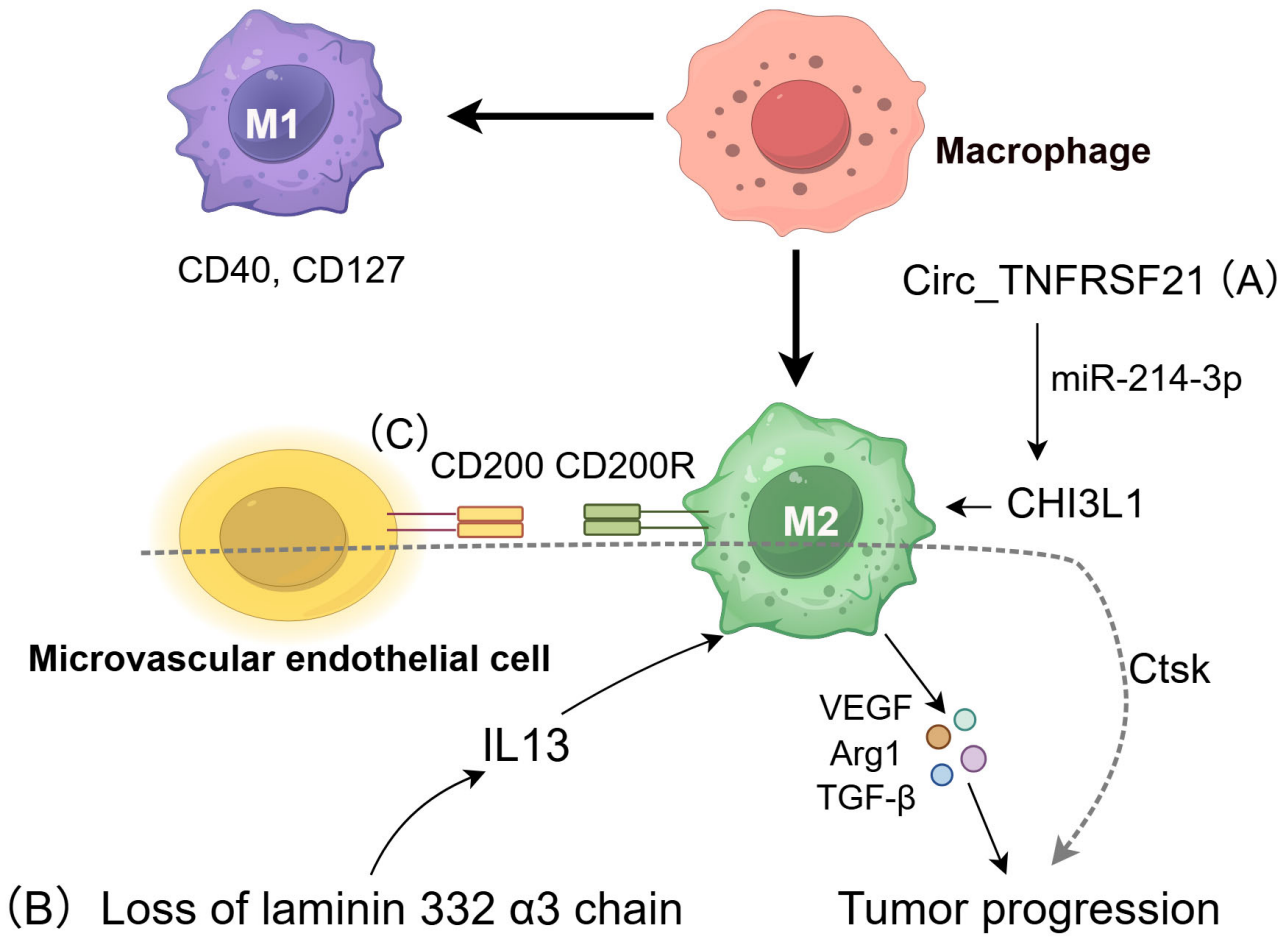
The role of macrophages in cSCC. TAM can regulate cSCC pathogenesis through divergent polarization states. Classically activated M1 macrophages suppress tumor growth through anti-cancer immunity, whereas alternatively activated M2 macrophages promote tumor progression via immunosuppressive cytokines (VEGF, Arg-1, TGF-β, IL-10) (21). M2 polarization is influenced by three critical pathways: **(A)** Circ_TNFRSF21 enhances M2 polarization through the miR-214-3p/CHI3L1 signaling pathway (30); **(B)** Loss of the laminin 332 α3 chain triggers IL-13 secretion, recruiting M2 macrophages (29); **(C)** CD200-CD200R interactions stabilize the M2 phenotype and drive invasion by upregulating cathepsin K (Ctsk) (32).

Prior research indicates that TAMs in cSCC show varied activation states, with macrophages undergoing dynamic polarization due to the tumor’s diverse microenvironments (25). These macrophages not only express functional M2-associated markers, such as Arg-1 and matrix metalloproteinase 9 (MMP9), but also significantly upregulate M1-associated markers, including CD40 and CD127 (26) (**Figure 1**). Despite the presence of M1 signals, the weak classical activation of macrophages, coupled with the substantial production of tumor-promoting growth factors, impedes the complete eradication of tumors (27). Moreover, increased M2 macrophages polarization was associated with higher aggressiveness and poorer prognosis in cSCC. The substantial infiltration of macrophages into the TME may facilitate tumor progression by promoting angiogenesis and tissue remodeling, as well as the production of lymphangiogenic factors, such as VEGF-C (28). Consequently, enhancing M1 activation in TAMs is a promising target for cancer therapy. Caley et al. identified the α3 chain of laminin 332 as a potential immunotherapy target in cSCC, linking its loss to high metastatic risk and elevated IL-13 secretion, which promotes M2 macrophages recruitment (29).

Recent research emphasizes the influence of non-coding RNA on macrophage polarization, with Circ_TNFRSF21 facilitating M2 polarization through the miR-214-3p/CHI3L1 pathway. By preventing cSCC proliferation and metastasis *in vivo*, Circ_TNFRSF21 knockdown advances our knowledge of the mechanisms underlying cSCC and raises the possibility that the Circ_TNFRSF21/miR-214-3p/CHI3L1 axis could be a useful therapeutic target or diagnostic marker (30).

CD200 is an established immunosuppressive protein that is predominantly found in the microvascular endothelial cells of cSCC. Its homologous receptor, CD200 receptor (CD200R), is mainly expressed in CD163^+^ macrophages and CD11c^+^ dendritic cells. The interaction between CD200 and CD200R on these immune cells inhibits pro-inflammatory activation, thereby maintaining macrophages in an M2 polarized state (31). Subsequent studies have shown that CD200 facilitates cSCC invasion and migration by inducing cathepsin K (Ctsk) expression in macrophages and DCs (32). *In vitro* studies have demonstrated that inhibition of the CD200-CD200R interaction and Ctsk can impede cSCC invasion and metastasis (33). These findings suggest that CD200 and Ctsk have a role in the immune evasion mechanisms of cSCC and could serve as promising therapeutic targets.

A recent study utilizing both *in vivo* and *in vitro* experiments revealed that activation of the neurotrophin receptor CD271 facilitates macrophage recruitment, potentially contributing to the suppression of tumor aggressiveness. These findings indicate that CD271 may serve as a promising therapeutic target for future drug development (34).

### 2.2 Neutrophils

Neutrophils, the most prevalent cells in the bloodstream, are among the first immune responders to damaged tissues. They combat pathogens by engulfing them, releasing antimicrobial proteins and protease, and creating neutrophil extracellular traps (NETs) (14). Besides fighting infections, neutrophils can invade cancer and participate in their progression, demonstrating significant phenotypic and functional plasticity (35). Similar to TAMs, neutrophils in the TME exhibit dual roles, with both anti-tumor and pro-tumor functions. Tumor-associated neutrophils (TAN) exhibit two phenotypes: the pro-tumor N2 phenotype, driven by TGF-β, and the anti-tumor phenotype (or N1), influenced by interferon-β (IFN-β) or TGF-β signaling suppression (36). Involvement of N2 TANs spans every phases of tumor progression, from its onset to metastasis and immune suppression (37). These cells have the capacity to enhance tumor proliferation, angiogenesis, and immunosuppression within the TME through mechanisms such as the release of cytokines, including neutrophil elastase (NE) and MMP-9, as well as the suppression of NK cell function. Conversely, N1 neutrophils can demonstrate anti-tumor activity by secreting cytotoxic mediators, such as reactive oxygen species (ROS), or through direct interactions with tumor cells (38, 39). Generally, a significant presence of neutrophils in solid tumors suggests a negative clinical outcome for patients (40). For example, poor survival rates in a variety of solid tumors, especially advanced malignancy, are associated with a high peripheral blood neutrophil-to-lymphocyte ratio (NLR) (41). Moreover, their roles as poor prognostic factors for cSCC have been confirmed in many studies recently (42–44). Emerging evidence indicates that TANs may impede cancer development by directly eliminating tumor cells or enhancing innate and adaptive immunity (45, 46).

Neutrophils are delicate cells with a short half-life *in vivo* (approximately 8 h) and are highly vulnerable *in vitro*, complicating the capture and study of their functions within the TME (47). Research on neutrophils in cancer has largely concentrated on animal models or the roles of circulating human neutrophils (48). Khou et al. explored how TANs contribute to the development of cSCC in animal models. Their study revealed a positive correlation between TAN infiltration and tumor volume, with TANs demonstrating immunosuppressive roles that hindered effector CD8^+^ T cell responses and facilitated tumor progression (49). However, additional clinical samples are required to validate these phenomena observed in mouse models.

Moeller and colleagues recently identified NETs in metastatic cSCC for the first time (50). They discovered that in cSCC, NET formation extends beyond the ulcerated areas and is linked to neutrophil aggregation. Earlier research has indicated that NETs can cause CD8^+^ T lymphocytes to exhibit an exhausted phenotype by attaching to the immunosuppressive ligand PD-L1, which tilts the TME in the direction of immunosuppression (51). In animal studies, DNase I-mediated removal of NETs enhances the effectiveness of anti-PD-1 treatment by boosting the infiltration of CD8^+^ T cell and increasing their cytotoxic activity (52). Therefore, NETs represent promising therapeutic targets. However, we still don’t fully grasp how NETs contribute to the evolution of cSCC, underscoring the need for further investigation into the specific mechanisms of NETs in tumor progression and exploration of intervention strategies that target NETs to develop novel therapeutic options.

Recent progress in single-cell sequencing technology have increasingly enabled studies to elucidate the functions and heterogeneity of tumor neutrophils at the single-cell level (53, 54). However, no study has provided a comprehensive atlas of neutrophils in the TME of patients with cSCC. The thorough application of single-cell multiomics approaches may open new directions for understanding neutrophil functions in the TME.

### 2.3 Dendritic cells

Originating from CD34^+^ hematopoietic stem cells located within the bone marrow, DCs are extensively distributed across the body (55). They comprise a diverse set of specialized cells that present antigens and are vital for activating and modulating both innate and adaptive immune responses (56). Conventional dendritic cells (cDCs), originating from common dendritic cell progenitors (CDPs), are categorized into two primary types: cDC1 and cDC2. DCs encompass various developmentally distinct cell types, such as monocyte-derived dendritic cells (MoDCs), plasmacytoid dendritic cells (pDCs), and Langerhans cells (LCs) (57). Tumor-associated cDCs are thought to capture dead tumor cells or debris and convey cancer antigens as peptide-MHC complexes to draining lymph nodes, facilitating T cell activation and initiation (58, 59).

**Figure 2:**
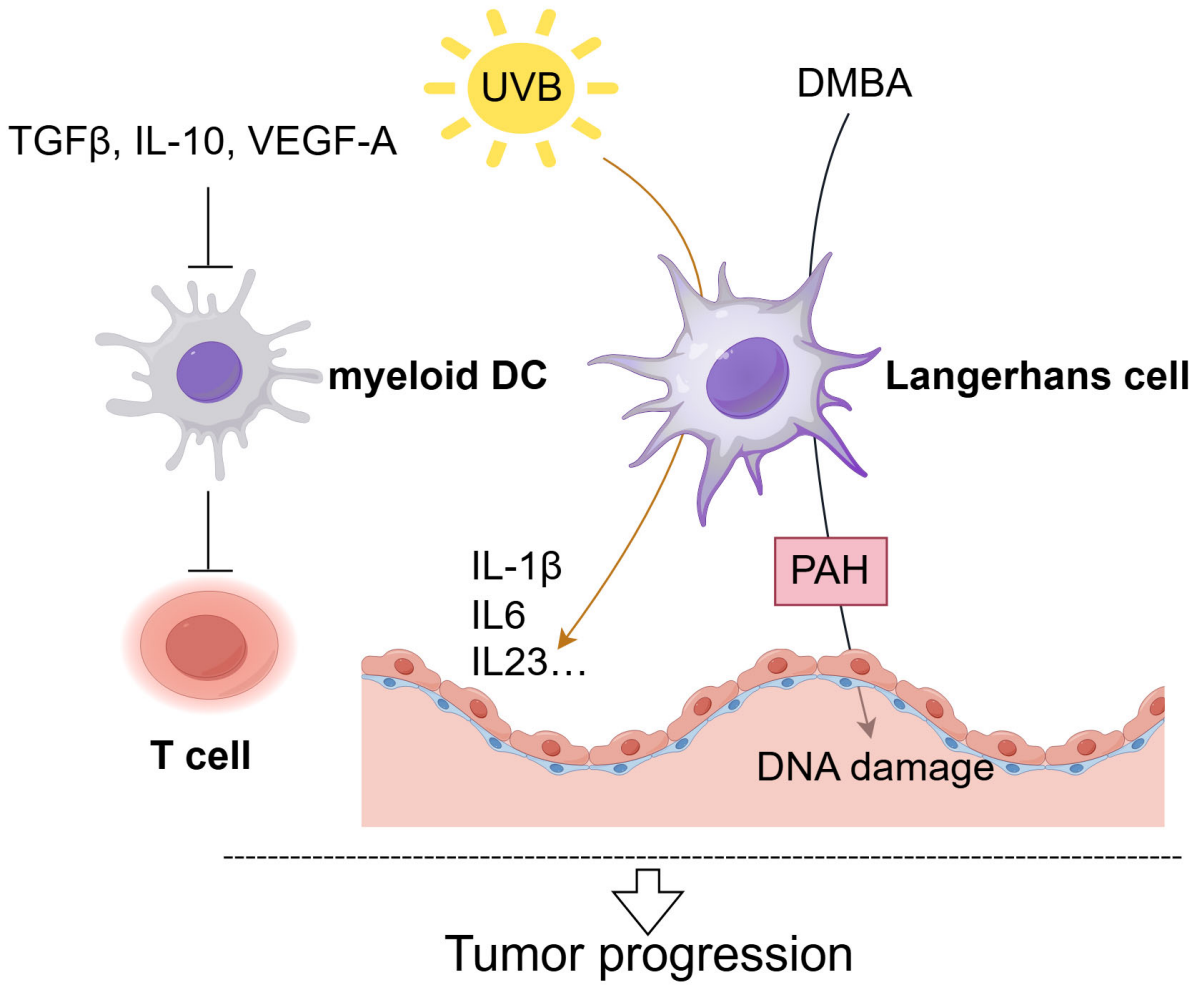
The role of DCs in cSCC. Langerhans Cells (LCs) critically drive cSCC progression. Exposure to excessive UVB radiation significantly reduces LC density and LC mainly promote tumor progression by upregulating pro-inflammatory cytokines (IL-1β, IL-6, IL-23) expression (66). When exposed to the carcinogen DMBA, LCs enhance epithelial DNA damage through polycyclic aromatic hydrocarbon (PAH) metabolism, facilitating squamous cell carcinoma development (68). And myeloid dendritic cells in cSCC demonstrate impaired T-cell activation, with their functionality further suppressed by factors including TGF-β, IL-10, and VEGF-A (63).

cSCC results from the malignant growth of epidermal KC, positioning LCs as the initial APCs to encounter tumor antigens (60). Fujita et al. showed that LCs in cSCC effectively induce type 1 immune responses *in vitro*, yet the patients’ immune systems frequently do not eliminate cSCC tumors (61). The comparatively limited numbers of LCs in TME provides a reasonable explanation for this inconsistency. Specifically, cSCC lesions exhibited a decrease in the number of LCs, CD11c^+^ DCs, and CD123^+^ pDCs (62, 63). Exposure to excessive UVB radiation, chemical carcinogens, or tumor promoters significantly reduces LC density (64, 65). Under UVB stimulation, LCs primarily exert their pro-tumor effects by enhancing the epidermal IL-1β, IL-6, IL-23, and NOS2 expression, along with raising the level of the epithelial growth factor IL-22 (66) (**Figure 2**). Qu et al. established that the depletion of LC and local immunosuppressive microenvironment are essential in the progression of skin cancer, linking LC depletion to tumor advancement (67). In animal models, Modi et al. found that under the stimulation of the carcinogenic agent 7,12-dimethylbenz anthracene (DMBA), LCs promote epithelial DNA damage as well as enhance chemical carcinogenesis through polycyclic aromatic hydrocarbon (PAH) metabolism, thereby facilitating the occurrence of squamous cell carcinoma (68). Additionally, the impairment of immune surveillance and the ability of LCs to promote regulatory T cells (Tregs) in the context of DNA damage contribute to skin cancer development (69). Moreover, high PD-L1 expression in DCs mediates immunosuppression in the TME by influencing the differentiation state of DCs and induce T cell anergy (70, 71). In contrast to LCs, myeloid DCs in cSCC are not efficient at stimulating T lymphocyte proliferation, and the existence of cytokines TGF-β, IL-10, and VEGF-A in the TME is thought to inhibit the function of myeloid DCs (63). Another notable feature of the cSCC microenvironment is the abundant presence of pDCs, which release IFN-α upon encountering foreign antigens, potentially contributing crucially to anti-tumor immune responses (63, 72). Overall, given their pivotal function integrating innate and adaptive immunity and trigger immunological responses, DCs present promising prospects as candidates for cancer immunotherapies.

### 2.4 Myeloid-derived suppressor cells

MDSCs represent a varied group of myeloid cells at various differentiation stages (73), comprising two major cell types: granulocytes or polymorphonuclear cells (PMN-MDSCs) and monocytes (M-MDSCs) (74). PMN-MDSCs resemble neutrophils in both phenotype and morphology, while M-MDSCs are akin to monocytes. The defining characteristic of these cells is their capacity to impede immune responses, thereby enabling tumor cells to evade recognition and elimination by the immune system (75). Evidence suggested that MDSCs can prevent the actions of T cell and NK cell by upregulating factors such as ARG1, ROS, TGF-β, IL-10, and PD-L1. Moreover, MDSCs aid in carcinogenesis and metastasis by supporting the survival of cancer cells, stimulating angiogenesis, and facilitating tissue remodeling (76). A poor prognosis is associated with high concentrations of MDSCs accumulating in several solid tumors (77). Moreover, a variety of findings have shown that MDSCs are crucial prognostic indicators for tumor advancement and potential targets for anti-cancer therapies (78).

However, studies on MDSCs in cSCC are limited. A study identified MDSCs as key NO producers in SCC, and inhibiting NO may restore the expression of vascular E-selectin, which may in turn improve the recruitment of T cells (79). As mentioned above, the populations of circulating and intra-tumoral neutrophils and/or G-MDSCs are elevated (43). Transmembrane glycoprotein CD147, which is a component of the immunoglobulin superfamily, shows substantial expression in various cancers. Research suggests that CD147 is crucial for malignant epidermal transformation and tumor initiation by activation of KC and the recruitment of MDSCs through the RSK2/AP-1 pathway (80). Furthermore, as previously mentioned, CD200 can induce CTSK to stimulate cSCC invasion and metastasis through the CD200-CD200R axis, with MDSCs and TAMs being the primary sources of Ctsk protein (32). Stumpfova et al. observed that CD200 serves as an indicator of SCC metastasis and that metastatic survival depends on CD200^+^ SCC KC’s capacity to directly interact with and control CD200R^+^ MDSCs (81). MDSCs, as crucial elements of the TME, have received increased attention for their impact on cancer progression and treatment response. Therefore, establishing MDSC subpopulations as therapeutic targets and biological markers that reflect the response to prevailing cancer treatments requires a fuller comprehension of the mechanisms underpinning MDSC genesis, recruitment, and functionality.

### 2.5 NK and ILCs

ILCs are derived from a common lymphoid progenitor (CLP), just like adaptive T and B cells, but they lack somatic rearrangement of antigen receptors and exhibit no antigen specificity (82). They are divided into five subsets: NK cells, lymphoid tissue inducer (LTi) cells, and three groups of ILCs, namely ILC1, ILC2, and ILC3. The three groups of ILCs demonstrate a resemblance to their corresponding helper T cell subtypes (Th1, Th2, and Th17 cells) and secrete cytokines that influence the immune system both innately and adaptively (83). ILCs are tissue-resident cells that sense microenvironmental changes and quickly secrete cytokines, acting as initial coordinators of immune responses (84). The biological function of ILCs, whether pro-tumor or anti-tumor, is largely depended on the tissue type and cytokine environment. For example, NK cells represent pivotal elements of intrinsic immunity, endowed with the capacity to elicit potent anti-tumor responses through direct cell death of neoplastic cells or the augmentation of antibody- and T cell-mediated reactions (85). Conversely, NK cells may express immune checkpoints like NKG2A, which suppresses their ability to fight tumors. Additionally, NK cells can transform into less effective anti-tumor ILC1s due to TGF-β signaling, where surface immune checkpoints and TGF-β accumulation within the TME encourage carcinogenesis (86). Similarly, type 2 ILCs have been demonstrated to possess the capacity to release IL-4, IL-5, and IL-13, which suppresses the immune system in the TME and exerts a direct effect on tumor cells to promote their growth and metastasis (87). Likewise, 17-type polarized ILC subtypes secrete IL-17A, which contribute to metastasis by facilitating angiogenesis through the stroma or stimulate tumor cells proliferation directly (88, 89). The functional plasticity of ILCs offers opportunities for the design of novel cancer immunotherapies.

**Figure 3:**
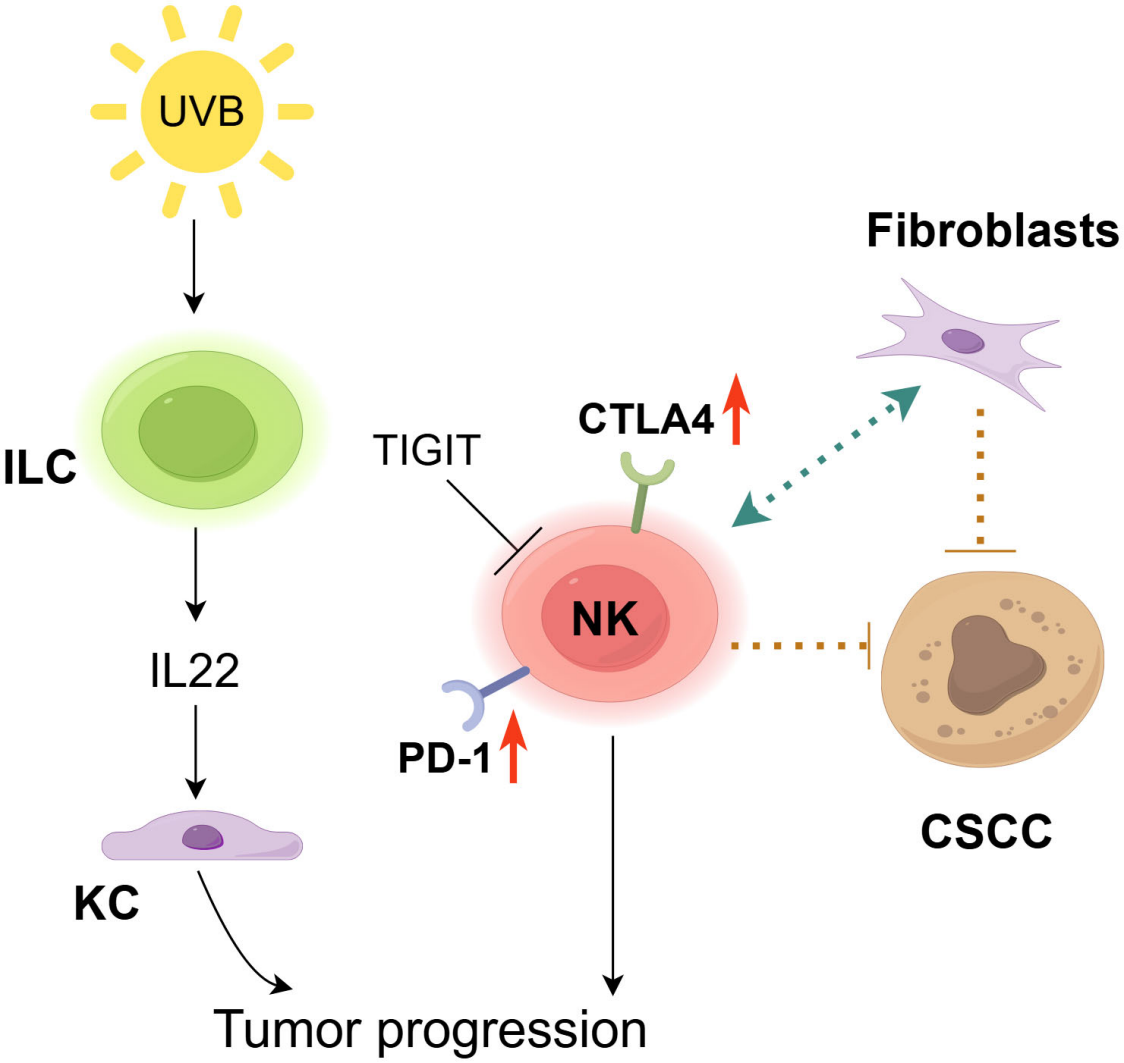
The role of NK cells in cSCC. NK cells exhibit a context-dependent dual role in the carcinogenesis of cSCC. On one hand, NK cells suppress cSCC growth by directly interacting with tumor cells and CLEC2A^+^ fibroblasts (91). However, inhibitory receptors (CTLA-4, PD-1) are upregulated in NK cells and ILC1s within the tumor microenvironment (92), while TIGIT induces NK cell exhaustion to promote tumor progression (93). Furthermore, chronic UV exposure expands IL-22-producing ILC3s, which drive keratinocyte (KC) clonal expansion, thereby accelerating tumor progression (95).

NK cells have been demonstrated to exhibit direct interaction with tumor cells (90) and CLEC2A-positive fibroblasts, thereby exerting a suppressive effect on the growth of cSCC (91). Furthermore, NK and Langerhans cells collaborate to impede the development of tumors in chemically induced mouse models of carcinogenesis (64). Through transcriptomic and immunophenotypic analyses, Luci et al. detected the presence of NK cells, ILC1s, and some ILC3s in mouse and human tumor tissues (**Figure 3**). They also found an increase in inflammatory ILC1s during the precancerous stage and observed compromised anti-tumor functions in both NK cells and ILC1s, potentially facilitating cSCC progression. During the developmental stages of cSCC, characterized by the presence of papilloma and tumor, there is an observed upregulation of traditional inhibitory receptors, such as CTLA4 and PD-1, in ILC1s and NK cells (92). TIGIT-induced NK cell depletion and the capacity to impede tumor progression by blocking TIGIT corroborate the assertion that the TIGIT-induced NK cell inhibitory axis is prominent in human cSCC (93). The results indicate a potential approach for cSCC immunotherapy. The proportion of NKp46 ILC1s in the premalignant lesion was significantly higher than that of NKp46 NK cells expressing these activating receptors in the tumor stage, suggesting ILCs’ significant involvement in the initial malignant transformation of KC (94). Additionally, Lewis and his colleagues revealed that long-term UV exposure induces phenotypically distinct ILC3 populations, which associate with p53^+^ KC islands and produce chemokines, such as IL-22, playing a crucial role in promoting KC clonal expansion (95). Therefore, a thorough analysis of ILCs in skin lesions is necessary to fundamentally understand their role in tumor progression, enabling the development of optimal immunotherapy approaches to prevent cSCC progression. Overall, there is a critical need to comprehend (i) the diversity within ILC subgroups, including their propensity to transdifferentiate into other ILC subgroups within tumors and (ii) the key role of tissue-derived cytokines in driving ILC-specific responses to effectively target these populations.

### 2.6 Innate-like T cells

Innate-like T cells (ILTCs), or unconventional T cells, comprise γδ T cells that detect phosphorylated antigens, invariant natural killer T (iNKT) cells with invariant αβ T cell receptors (TCRs) recognizing glycolipid antigens via CD1, and mucosal-associated invariant T (MAIT) cells that identify riboflavin-derived antigens with MR1 (96, 97). These cells exhibit pleiotropic functions and exhibit a rapid response to non-peptide antigens through conserved TCRs. Similar to ILCs, ILTCs are lymphocytes that reside in tissues and are found abundantly at many tumor sites. Through homologous receptors, they detect cytokines and alarmins, prompting a rapid release of factors that protect tissues or promote inflammation, thus serving as first responders in the TME (98, 99).

The growth of tumors is influenced by ILTCs, which can function in both pro-tumor and anti-tumor capacities. In an environment induced by type 1 cytokines (mainly IL-12 and IL-15), they can mediate anti-tumor effects through granzymes and perforin or antibody-dependent cell cytotoxicity (ADCC) (98, 100). ILTCs can also enhance cytotoxicity through TCR signaling, thereby indirectly killing tumors by secreting IFNγ (101). Additionally, NKT cells stimulate DC maturation through CD40-CD40L interactions, enabling DCs to effectively present cancer-derived antigens to CD8^+^ T cells, thereby enhancing tumor-specific immunological responses (102). Research indicates that the number and metabolic activity of NKT and γδ T cells within tumors or in circulation correlate with favorable prognoses across various cancer types (102, 103). A hostile TME, however, can exploit ILTCs’ abilities in tissue repair and homeostasis, steering them towards promoting tumors (86). Like ILCs, type 2 primed ILTCs encourage cancer growth and spread, while type 17 polarized ILTC release IL-17A, enhance angiogenesis, and promoting tumor growth (14). The disruption of TGF-β expression within the TME is pivotal to tumor evasion of the immune system and poor response to anti-tumor treatments. Furthermore, NK cells can become less cytotoxic when transform into ILC1 and ILC1-like cells, thus failing to effectively regulate tumor proliferation and dissemination. Additionally, the TGF-β released by ILTCs can further promote an immunosuppressive microenvironment (104).

**Figure 4:**
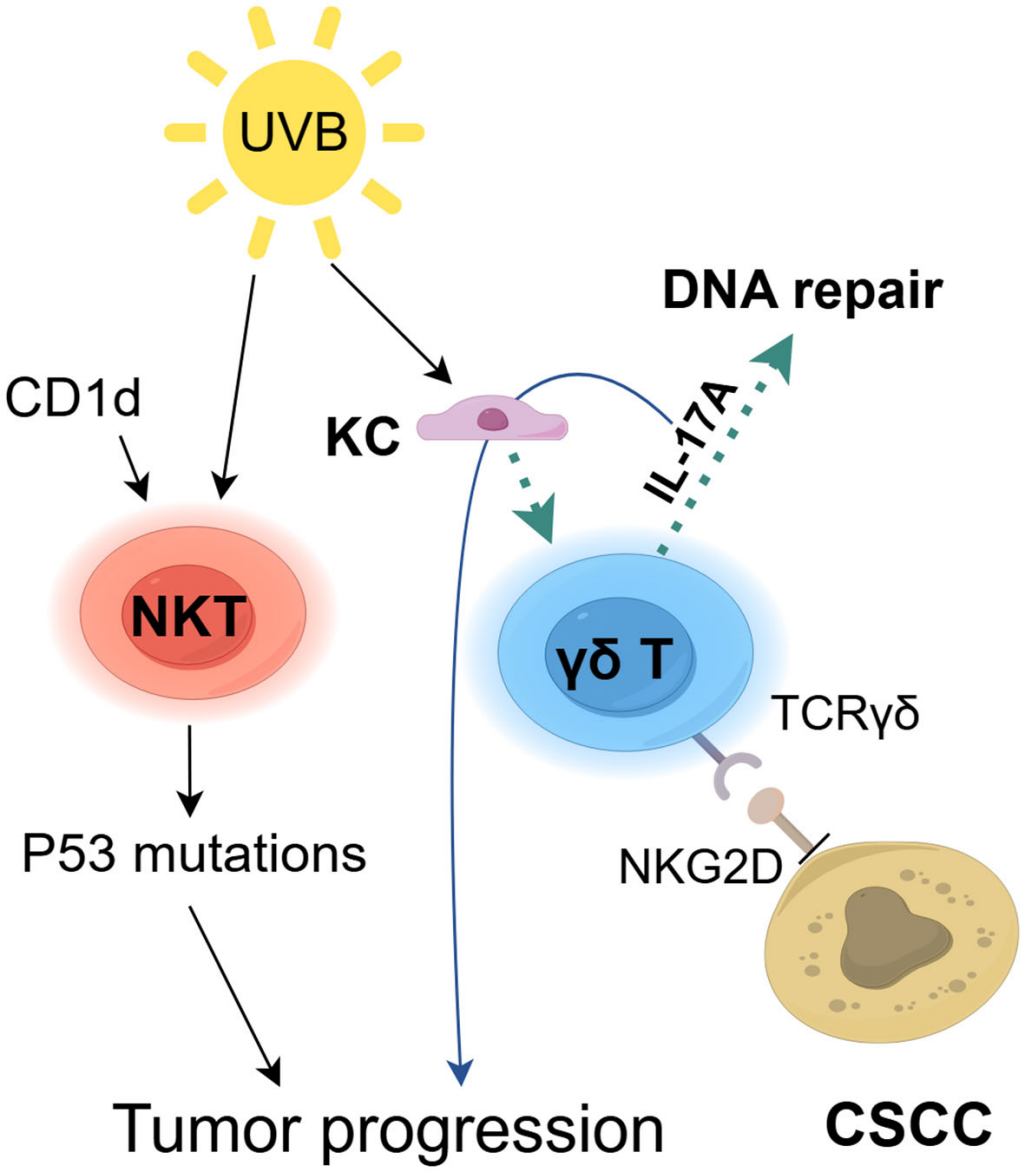
The role of ILTCs in cSCC. Innate-like T cells (ILTCs) exhibit dual pro-tumor and anti-tumor functions in cSCC pathogenesis. While they directly kill SCC cells through surface receptors TCRγδ and NKG2D (106), UV-damaged keratinocytes (KC) activate γδ T cells to secrete IL-17A, which paradoxically enhances DNA repair mechanisms (108) but simultaneously stimulates epithelial hyperproliferation to drive tumorigenesis (109). Furthermore, the CD1d-NKT cell axis promotes UVB-induced p53 mutations and immunosuppression, synergistically accelerating cSCC progression (111).

Dendritic epidermal T cells (DETCs), a type of γδ T cell located in the mouse epidermis, serve as the primary anti-tumor participants in this tissue (105). DETCs rely on the binding of γδ T cell surface receptors, TCRγδ and NKG2D, to kill SCC cells (106) (**Figure 4**). Another study suggests that γδ T cells provide protection against chemically induced SCC (107). Additionally, UV-damaged KC trigger DETCs to produce IL-17A, which upregulates the molecules involved in DNA repair responses (108). Therefore, DETCs may play a role in the prevention of UV-induced skin cancer, although additional research is required. Nevertheless, IL-17A can also stimulate the rapid growth of skin epithelial cells, thereby promoting tumorigenesis (109). Our knowledge of DETC biology remains limited, especially regarding DETC responses to the skin TME (110). Exploring the anti-tumor mechanisms of DETCs could provide valuable insights for research on human epidermal γδ T cells. NKT cells exert pro-cancer effects in UVB-induced cSCC. The CD1d-NKT cell axis contribute significantly to the promotion of UVB-mediated p53 mutations, immunosuppression, and skin tumor development, whereas CD1d knockout reduces UVB-induced processes such as inflammation, tumorigenesis, and the absence of functional NKT cells (111, 112). However, their contribution to the progression of human cSCC is still uncertain. In conclusion, thoroughly investigating ILCs in skin lesions is necessary to fundamentally understand their role in tumor progression, enabling the development of optimal immunotherapy approaches to prevent cSCC progression.

### 2.7 Mast cells

Mast cells (MCs) are essential components of immune systems and are involved in conditions such as autoimmune disease, cardiovascular disease and allergy (113). Originating from specific bone marrow progenitor cells, MCs are part of the innate immune system. They migrate to and mature in tissues in response to specific microenvironmental conditions (114). Depending on their function, the locations of MCs in human tissues vary, although they are primarily abundant near blood vessels, epithelial cells, fibroblasts, and nerves (115). Although the roles of MCs in allergic and parasitic reactions are well characterized, their involvement in carcinogenesis is not fully elucidated. Although they are not an insignificant population in the TME, the involvement of MCs in cancer is controversial. MCs may be found at the tumor margin or within infiltrating tumors, and it has been reported that, depending on their abundance, location, stimuli, and the tumor environment, MCs can exhibit either pro-tumor or anti-tumor characteristics (116, 117). MCs commonly accumulate in tumors and adjacent tissues across various tumor types. Recruited to the TME by a variety of cytokines, MCs are regulated by extracellular vesicles (EVs) or active substances released from cancer cells or by interacting directly with tumor cells (113, 118). Once activated and degranulated, MCs become intensely pro-inflammatory, recruit innate immune cells (mainly neutrophils, macrophages, and eosinophils) as well as adaptive immune cells (B and T cells) to coordinate anti-tumor immune responses (119, 120). However, MCs can also participate in tumor development by synthesizing and storing angiogenic factors and matrix metalloproteinases, which enhance tumor angiogenesis and invasion, respectively (121). Mast cells can also induce immune suppression through secreting IL-10, histamine, and TNF-α (122, 123).

MCs have been shown to exhibit an immunosuppressive phenotype in skin cancer but a pro-inflammatory phenotype in chronic skin inflammation, with the potential mechanisms and transition pathways remaining unclear (124). Medler and colleagues utilized a K14-HPV16 transgenic mouse model and demonstrated that C5a mediates C5aR-dependent MC activation in SCC, potentially influencing their suppressive impact on CD8^+^ T cell cytotoxicity. Additionally, C5aR1^−/−^ mice are less prone to developing tumors (125). UVB stimulates vitamin D3 synthesis, which is released by MCs via the vitamin D receptor expressed on its surface. Vitamin D3 exerts immunosuppressive effects by promoting IL-10 release (126). Another potential mechanism by which MCs promote SCC progression is through the expression of CYP27A1 and CYP27B1, which synthesize calcitriol (a metabolite of vitamin D3 known for its immunosuppressive properties), thereby inhibiting IgE-dependent MC activation (127). These results offer new insights and directions for exploring future cancer immunotherapies targeting MCs.

### 2.8 Innate immune cells as therapeutic targets for cSCC

Considering the crucial involvement of innate immune cells in controlling cSCC progression and orchestrating anti-tumor responses, there has been growing interest in therapeutic strategies targeting innate immunity. Numerous preclinical investigations underscore the potential of targeting innate immune cells, with strategies including reprogramming, depletion, or reduction of immunosuppressive cells demonstrating promise as therapeutic approaches. While a number of clinical trials aimed at innate immune cells are currently underway, clinical trials specifically focusing on cSCC have yet to be initiated. In this context, we summarize the preclinical studies related to innate immunity in cSCC to provide directions for future clinical research (**Table 1**).

**Table 1:** Therapeutic strategies targeting innate immunity in cSCC.

Immune Cells	Therapeutic Targets	Targeted Therapies	Mechanisms and Functions
Macrophages	Macrophages	Alkannin	Enhance cellular apoptosis and induce M1 macrophage polarization through the upregulation of PTEN
	Macrophages	Methionine Enkephalin	Decrease the population of MDSCs and modulate the polarization of TAMs
	Macrophages	Imidazoquinoline	Polarize the macrophage towards a Th1 and M1 cytokine profile
	Macrophages	ALA-PDT	induce the expression of CCL8 and recruit M1 macrophages
	Macrophages	Nanoparticles encapsulating IL-12	Facilitate the conversion of macrophages from the M2 to the M1 phenotype
	Arginase	Arginase inhibitor	Attenuate immune suppression
Neutrophil	COX-2	Celecoxib	Mitigate neutrophil infiltration and activation and the synthesis of prostaglandin E2
	CXCR2	CXCR2 inhibitors	Impede neutrophil recruitment and reduce the formation of NETs
Myeloid-Derived Suppressor Cells	NO	iNOS inhibitors	Augment anti-tumor immune responses
	MDSC	Combined therapy of all-trans retinoic acid and CTLA-4 blockers	Reduce circulating MDSC numbers, enhance treatment response rates and effectiveness
Dendritic Cells	DC	Combining autologous CD16^+^ DC vaccination and anti-PD-L1 antibody with radiotherapy	Enhance T cell-mediated anti-tumor efficacy
	DC	Methionine Enkephalin	Augment DC activation by inducing autophagy and facilitating the release of DAMPs
ILCs and ILTCs	γδ T cells	Activin inhibitors	Promote proliferation of epidermal γδ T cells, decrease skin tumor formation and malignant progression
	NKT	CD1d knockout	Reduce UVB-induced inflammation, carcinogenesis, and the absence of functional NKT cells
Mast cells	VEGF-C, VEGF-D	VEGF-C and VEGF-D inhibitors	Block MC-derived pro-angiogenic mediators
	CXCR4, CXCL12	AMD3100	Disrupt migration of MCs to draining lymph nodes

TAMs, Tumor Associated Macrophages; ALA-PDT, 5-aminolevulinic acid-mediated photodynamic therapy; COX-2, Cyclooxygenase-2; MDSCs, Myeloid-Derived Suppressor Cells; iNOS, Inducible nitric oxide synthase; DCs, Dendritic Cells; DAMPs, Damage-Associated Molecular Patterns; ILCs, Innate lymphoid cells; ILTCs, Innate-like T cells; MCs, Mast cells.

A potential approach for targeting macrophages involves leveraging their inherent plasticity. Research indicates that alkannins can impede cSCC growth by promoting cell apoptosis and the polarization of M1 macrophages through the upregulation of PTEN (128). Furthermore, methionine enkephalin mitigated immune suppression by decreasing the population of MDSCs and polarizing TAMs to M1 phenotype *in vivo* (129). In a similar manner, the administration of local imidazoquinoline has been demonstrated to shift the macrophage population in cSCC towards a Th1 and M1 cytokine profile, thereby decelerating tumor progression (130). Additionally, 5-aminolevulinic acid-mediated photodynamic therapy (ALA-PDT), a non-invasive or minimally invasive treatment for cSCC, induces the expression of CCL8 and recruits M1 macrophages, consequently inhibiting tumor growth (131). However, M1 macrophages exhibit the capacity to readily transition back to the M2 phenotype upon stimulation, necessitating sustained M1 differentiation to counteract this tendency (132). The encapsulation of IL-12 in nanoparticles has been shown to facilitate the transition of macrophages to M1 phenotype within the TME, effectively protecting against melanoma development in murine models (133). As stated previously, M2 macrophages secrete arginase, which contributes to immune suppression. A recent investigation employing a preclinical immune-privileged mouse model of cSCC showed that the localized inhibition of arginase significantly diminishes cSCC tumorigenesis. This effect is particularly pronounced when combined with checkpoint inhibitors, thereby providing promising prospects for the future development of localized adjuvant therapies for cSCC (134).

Cyclooxygenase-2 (COX-2) is an enzyme implicated in inflammatory processes, mainly by synthesizing prostaglandins, and is associated with the pathogenesis of cSCC (135). Empirical evidence indicates that applying the COX-2 inhibitor celecoxib topically after UVB exposure successfully reduces neutrophil infiltration, activation, and prostaglandin E2 production (136). Furthermore, the topical administration of celecoxib decreases chronic inflammation and inhibit the forming of UVB-induced papillomas and carcinomas (137). These data strongly support the clinical potential of using topical COX-2 inhibitors for the prevention of human skin cancer. CXCR2, a critical receptor for neutrophil chemoattraction, is essential for integrins activation and neutrophils recruitment (138). Clinical trials designed to impede neutrophil recruitment through the disruption of CXCR1/2 signaling pathways have already commenced (40). Targeting CXCR2 has been shown to significantly reduce NET formation in melanoma, and inhibition of NET formation has been observed to enhance tumor sensitivity to double checkpoint blockade using PD-1 and CTLA-4 inhibitors (51).

MDSCs exert direct immunosuppressive effects and facilitate the proliferation of additional immunosuppressive cell populations, including Tregs and TAMs, thereby perpetuating the immunosuppressive TME (139). Strategic targeting of MDSCs to selectively and effectively eradicate these immunosuppressive elements within the tumor milieu is a promising avenue for therapeutic intervention. Pharmacological agents that inhibit NO production, including inducible nitric oxide synthase (iNOS) inhibitors, have the potential to treat cSCC and its precancerous lesions, such as actinic keratosis, by augmenting anti-tumor immune responses (79). A considerable body of preclinical and clinical research has evaluated the safety and efficacy of MDSC inhibition, both as a monotherapy and in conjunction with other therapeutic modalities, to improve anti-tumor responses and address resistance mechanisms in cancer cells (140, 141). For example, in patients with melanoma, combination therapy with all-trans retinoic acid and CTLA-4 blockers has been shown to reduce circulating MDSC numbers (142). Thus, combining immunotherapy with targeting MDSCs may enhance treatment response and effectiveness in other skin cancers.

DCs are important cells that present antigens, crucial for activating T-cells and eliciting tumor-specific immune responses (143). Therapeutic strategies aimed at augmenting the immunogenic function of DCs have successfully triggered anti-tumor immune reactions in cancer patients. For instance, a therapeutic regimen combining autologous CD16^+^ DC vaccination and anti-PD-L1 antibody with radiotherapy demonstrated enhanced T cell-mediated anti-tumor efficacy and tumor size reduction in a patient with psoriasis and cSCC (144). Beyond its role in modulating MDSCs, methionine enkephalin has been shown to augments DC activation in cSCC by the induction of autophagy and the facilitation of damage-associated molecular patterns (DAMPs) release (145). These findings emphasize the crucial function of DCs in orchestrating both innate and adaptive immunity and underscore the substantial therapeutic promise of targeting DCs.

ILCs and ILTCs play crucial roles as primary responders within the TME during tumor initiation, immune surveillance, and progression. Their ability to swiftly integrate and respond to environmental cues makes them promising candidates for immunotherapeutic interventions (84). For example, research by Adhikary et al. indicated that hu man NK cells expanded ex vivo can effectively inhibit the oncogenic characteristics of cSCC cells, leading to a reduction in tumor growth. This study specifically found that NK cell therapy impedes the formation, invasion, viability, and growth of cSCC cell spheroids, indicating its potential utility as a therapeutic approach for cSCC (146). Additionally, activin, a critical factor in wound healing, was proven to prevent the proliferation of epidermal γδ T cells, thereby facilitating skin tumor formation and malignant progression. Consequently, activin inhibition has shown promise as a cancer therapy approach (147). Nonetheless, it is only in recent times that ILCs and ILTCs have been identified as pivotal components in cancer treatment. Consequently, immunotherapeutic strategies aimed at these cells remain in the nascent stages of development and require further investigation to evaluate their viability as innovative targets for cancer treatment.

MCs accumulate near tumor cells before angiogenesis begins, and tumor progression is closely associated with neovascularization. Thus, focusing on MC activation or inhibiting pro-angiogenic mediators from MCs might be an effective approach to prevent skin tumor development. Prior research has demonstrated that using soluble inhibitors to block the lymphangiogenic factors VEGF-C and VEGF-D results in fewer squamous cell tumors in transgenic mice (148). Furthermore, studies have demonstrated that the expression of CXCR4 on MCs and its ligand CXCL12, expressed by lymph node B cells, are crucial in the UV-induced migration of MCs to draining lymph nodes (149). The team led by Sarchio applied AMD3100, a CXCR4 antagonist, to interfere with this trafficking process and noted a marked decrease in skin tumor development (150).

## 3 Conclusion

Immunotherapy has fundamentally transformed cancer treatment, particularly through strategies that target adaptive immunity, like immune checkpoint inhibition and CAR T-cell therapy, which have demonstrated considerable promise in specific instances. Nevertheless, the general response rates are still low, underscoring the necessity of developing innovative therapeutic approaches. Comprehensive investigations of the TME in various cancers such as cSCC have elucidated the intricate and dynamic interactions between neoplasms and the host’s immune cells. These scientific advancements are revolutionizing immunotherapy, fundamentally transforming the therapeutic paradigm for metastatic cancers, and offering new therapeutic options for advanced and metastatic cSCC.

Most immune system components play dual roles, contributing to both the promotion and inhibition of tumors. This underscores the intrinsic plasticity of the innate immune system that can be strategically exploited for therapeutic interventions. Nonetheless, more studies are needed to elucidate the molecular determinants that reprogramming inflammatory cells towards an anti-tumor phenotype to effectively capitalize on this potential. Although treatments aimed at specific components of innate immunity have demonstrated potential, the development of more precise and efficacious strategies may require intricate combinations. These combinations should not only augment the inflammatory phenotype of immune cells, but also selectively inhibit or deplete immunosuppressive cytokines and cell types. Importantly, targeting immunosuppressive cells should avoid widespread elimination of all innate immune cells within the TME, as this could adversely affect the host. Considering the distinct characteristics of SCC in individual patients, evaluating these attributes prior to treatment is imperative to devise an optimal therapeutic regimen that enhances response rates while minimizing toxicity. Furthermore, strategies targeting the innate immune system must meticulously account for potential off-target effects and the risk of inducing excessive systemic inflammation. The identification of reliable biomarkers to predict patient responses to treatments based on innate immunity, along with the identification of optimal combination strategies, will constitute critical challenges in future research.

Significant advancements in emerging technologies in the field of immunomics have facilitated an unprecedented detailed examination of tumor immunity. Single-cell technologies enable in-depth analysis of immune cell subpopulations and spatial structures, thereby providing a more comprehensive understanding of the TME. Furthermore, artificial intelligence tools, including radiomics and deep learning models derived from digital pathology, have demonstrated considerable efficacy in predicting responses to immunotherapy (151). The incorporation of these technologies has markedly improved our capacity to predict drug efficacy and has accelerated the progress of developing novel therapeutic strategies. The integration of such innovations presents substantial potential for addressing the existing limitations in our understanding of innate immunity, potentially yielding transformative benefits for patients and heralding a new era in cancer immunology.

In summary, innate immune cells are vital for detecting and eradicating cancer cells as well as modulating adaptive immunity, thereby establishing a robust platform for novel immunotherapy development. The synergistic activation of both innate and adaptive immune responses presents significant potential for the advancement of cancer immunotherapy. By utilizing contemporary technologies to comprehensively analyze the intrinsic immune microenvironment of tumors, more effective and personalized treatment strategies can be formulated. These advancements are expected to propel the progress of cancer immunotherapy and influence its future.
